# Survival After Cancer Treatment at Top-Ranked US Cancer Hospitals vs Affiliates of Top-Ranked Cancer Hospitals

**DOI:** 10.1001/jamanetworkopen.2020.3942

**Published:** 2020-05-26

**Authors:** Daniel J. Boffa, Katherine Mallin, Jeph Herrin, Benjamin Resio, Michelle C. Salazar, Bryan Palis, Matthew Facktor, Ryan McCabe, Heidi Nelson, Lawrence N. Shulman

**Affiliations:** 1 Section of Thoracic Surgery, Department of Surgery, Yale School of Medicine, New Haven, Connecticut; 2 American College of Surgeons Cancer Programs, National Cancer Database, Chicago, Illinois; 3 Cancer Outcomes Public Policy and Effectiveness Research Center, Department of Internal Medicine, Yale School of Medicine, New Haven, Connecticut; 4 Section of Cardiovascular Medicine, Department of Internal Medicine, Yale School of Medicine, New Haven, Connecticut; 5 Department of Thoracic Surgery, Geisinger Heart Institute, Danville, Pennsylvania; 6 American College of Surgeons Cancer Programs, Chicago, Illinois; 7 Abramson Cancer Center, University of Pennsylvania, Philadelphia

## Abstract

**Importance:**

Hospital networks formed around top-ranked cancer hospitals represent an opportunity to optimize complex cancer care in the community.

**Objective:**

To compare the short- and long-term survival after complex cancer treatment at top-ranked cancer hospitals and the affiliates of top-ranked hospitals.

**Design, Setting, and Participants:**

This cohort study was conducted using data from the unabridged version of the National Cancer Database. Included patients were individuals 18 years or older who underwent surgical treatment for esophageal, gastric, lung, pancreatic, colorectal, or bladder cancer diagnosed between January 1, 2012, and December 31, 2016. Patient outcomes after complex surgical procedures for cancer at top-ranked cancer hospitals (as ranked in top 50 by *US News and World Report*) were compared with outcomes at affiliates of top-ranked cancer hospitals (affiliation listed in American Hospitals Association survey and confirmed by search of internet presence). Data were analyzed from July through December 2019.

**Exposures:**

Undergoing complex cancer treatment at a top-ranked cancer hospital or an affiliated hospital.

**Main Outcomes and Measures:**

The association of affiliate status with short-term survival (ie, 90-day mortality) was compared using logistic regression, and the association of affiliate status with long-term survival was compared using time-to-event models, adjusting for patient demographic, payer, clinical, and treatment factors.

**Results:**

Among 119 834 patients who underwent surgical treatment for cancer, 79 981 patients (66.7%) were treated at top-ranked cancer hospitals (median [interquartile range] age, 66 [58-74] years; 40 910 [54.9%] men) and 39 853 patients (33.3%) were treated at affiliate hospitals (median [interquartile range] age, 69 [60-77] years; 19 004 [50.0%] men). In a pooled analysis of all cancer types, adjusted perioperative mortality within 90 days of surgical treatment was higher at affiliate hospitals compared with top-ranked hospitals (odds ratio, 1.67 [95% CI, 1.49-1.89]; *P* < .001). Adjusted long-term survival following cancer treatment at affiliate hospitals was only 77% that of top-ranked hospitals (time ratio, 0.77 [95% CI, 0.72-0.83]; *P* < .001). The survival advantage was not fully explained by differences in annual surgical volume, with both long- and short-term survival remaining superior at top-ranked hospitals even after models were adjusted for volume.

**Conclusions and Relevance:**

These findings suggest that short- and long-term survival after complex cancer treatment were superior at top-ranked hospitals compared with affiliates of top-ranked hospitals. Further study of cancer care within top-ranked cancer networks could reveal collaborative opportunities to improve survival across a broad contingent of the US population.

## Key Points

**Question:**

Are there differences in survivorship between top-ranked cancer hospitals and affiliates that share a top-ranked hospital’s brand?

**Findings:**

In this cohort study of 119 834 patients who underwent surgical treatment for esophageal, gastric, lung, pancreatic, colorectal, and bladder cancer, risk of 90-day mortality after complex cancer treatment was higher and long-term survival was inferior at affiliate hospitals.

**Meaning:**

These findings suggest that quality improvement efforts are needed to address important differences in survival between top-ranked cancer hospitals and brand-sharing affiliate hospitals.

## Teaser

This cohort study examines short-term survival outcomes among patients who receive cancer treatment at top-ranked cancer hospitals compared with those who receive cancer treatment at affiliate hospitals.

## Introduction

Hospital selection can be a major factor in survival for a patient with cancer. Receiving complex cancer treatment from an insufficiently prepared hospital (eg, adequate experience, specialty trained surgeons) can expose a patient to a 4-fold increase in risk of death from surgical complications.^1,2,3^ Cure rates after cancer treatment are similarly variable,^4^ and several hospital characteristics (eg, higher volume hospitals, affiliation with a medical school) are associated with superior long-term survival.^5,6^ Therefore, patients with cancer have a critical need for information regarding the safety and quality of hospital care.

A hospital’s reputation for safety and quality (ie, the hospital’s brand) is a major consideration as patients evaluate hospitals for cancer care.^7,8^ National ranking systems, such as those published in *US News and World Report* (*USNWR*),^9^ can support hospital reputation for safety and quality. Such national ranking systems can influence patients’ hospital choice,^10,11^ and ultimately affect hospital market share.^12^

Top-ranked cancer hospitals have increasingly shared their brands with unranked hospitals through networks of affiliations.^13^ This brand-sharing has the potential to influence patients’ hospital preferences. In a recent nationally representative survey in the US, half of respondents believed that the safety, quality, and cure rates of complex cancer treatment would be the same at top-ranked hospitals and their affiliates.^14^ Furthermore, respondents indicated that affiliation with a top-ranked cancer hospital would increase their preference for a local hospital.^14,15^ At the same time, a 2019 study of Medicare enrollees^13^ found that mortality after complex surgical procedures to treat cancerwas 1.4-fold higher at affiliate hospitals compared with the top-ranked hospitals whose brands they shared.

We sought to examine short- and long-term outcomes across hospitals participating in top-ranked networks among patients 18 years or older. Quality as well as short-and long-term survival after complex surgical procedures to treat cancer were compared between top-ranked hospitals and affiliates of top-ranked hospitals (hereafter, *affiliates*) in the National Cancer Database (NCDB). Our objective was to establish a baseline differential of outcomes to inform future quality improvement efforts that leverage the network infrastructure and potential collaborations between programs.

## Methods

### Database

The National Cancer Database (NCDB) is a prospective registry of cancer care occurring at Commission on Cancer (COC)–accredited hospitals, capturing 72% of newly diagnosed malignant tumors in the US.^16,17^ A study-specific data set, deidentified at the patient and hospital levels, was created by the NCDB research team from the unabridged NCDB (eAppendix in the Supplement). No identifiers (eg, hospital name, location) were shared outside the NCDB. This study was approved by the Yale University institutional review board, and informed consent was waived owing to use of deidentified data. This study is reported following the Strengthening the Reporting of Observational Studies in Epidemiology (STROBE) reporting guideline.

### Hospitals

Hospitals that were ranked as being in the top 50 cancer hospitals by *USNWR* at least once between 2013 and 2016 were eligible to be analyzed as the top-ranked cohort. The *USNWR* rankings were chosen because they are widely recognized by the public, have been shown to influence patient choice,^8,10^ and potentially have the highest correlation with quality among publicly available indicators.^18^ Of note, there is approximately 75% concordance between designation by National Cancer Institute, and the *USNWR* top 50 cancer hospital status. Brand-sharing affiliates of top-ranked hospitals were identified as hospitals that (1) were affiliates of 1 of the top-ranked hospital in the American Hospital Association’s annual survey and (2) included the name of the top-ranked hospital (ie, the top-ranked hospital’s brand) in their web presence, as described previously.^13^ Both top-ranked hospital cohort and the affiliate cohort performing complex surgical procedures to treat cancer were further restricted to only those accredited by the COC.

### Patients

Patients 18 years and older who had undergone a complex surgical procedure as treatment for colorectal, lung, pancreatic, gastric, esophageal, or bladder cancer between January 1, 2012, and December 31, 2016, were eligible. The complex surgical procedures to treat cancer included colectomy (total or partial), proctectomy, esophagectomy, pulmonary lobectomy, pneumonectomy, pancreaticoduodenectomy (Whipple), gastrectomy (total or partial), and cystectomy. Surviving patients with less than 90 days of follow up (5.5% of patients) were excluded from the main analysis, but they were included in sensitivity analysis.

These complex procedures were chosen based on 2 perspectives. First, we wanted to focus on procedures with an operative mortality that exceeded 2%, a metric previously described as an indicator of high-risk cancer treatment.^19^ At the same time, we wanted to consider procedures involving different anatomic regions, and thus likely different surgical teams. Combined, the studied procedures represent more than 80% of complex cancer treatments in the US and are similar to those that have previously been studied as complex cancer treatments.^20,21^

### Variables

The primary independent variable was hospital type, coded as whether the surgical procedure was performed at an affiliate hospital. Additional covariates were patient age (ie, 18-44, 45-54, 55-64, 65-74, 75-84, or ≥84 years), sex, modified Charlson-Deyo comorbidity score (condensed to 0, 1, 2, or ≥3), year of diagnosis, administration of chemotherapy or radiation, pathological stage (*American Joint Cancer Committee, Seventh edition*^22^), tumor grade, insurance type (ie, commercial, no insurance or Medicaid, or Medicare), median income quintiles by zip code, and history of a prior malignant tumor (included as an important adjuster based on our prior work^23^). Several of the procedures had variations that involved removing surrounding structures, so an *extended* modifier was added to denote additional complexity in case one cohort performed more complex procedures than the other.

Additional variables were abstracted for descriptive purposes, including race/ethnicity, clinical stage, distance traveled to hospital, hospital census division, urban or metropolitan hospital location, and mean annual surgical volume. Variables and outcomes are further described in the NCDB data dictionary.^16,24^

The primary outcomes were time from diagnosis to treatment (ie, time-to-treat, calculated internally by NCDB), adherence to COC quality metrics, surgical margins, 30-day unplanned readmission, 90-day mortality (calculated from time of surgical procedure), and survival from the date of diagnosis (as opposed to surgical procedure date, as use of neoadjuvant treatment was variable). For survival analysis, patients were censored at death or day of last contact in the NCDB. The NCDB only captures readmissions in which the patient was readmitted to the same hospital where the surgical procedure was performed (ie, if the patient was admitted to a different hospital, the NCDB would not capture the readmission). Because patients are less likely to be readmitted to the hospital that performed surgical treatments as the travel distance increases, the analysis of readmissions was restricted to patients in both cohorts who traveled less than 50 miles for cancer treatment. For the analysis of the number of days between diagnosis and treatment (ie, time-to-treat), only patients in whom surgical treatment was the first treatment were analyzed. This restriction was imposed because induction chemotherapy and radiation regimens may vary in duration and toxic effects and could obscure a time-to-treat calculation

### Statistical Analysis

Patient demographic, clinical, and treatment characteristics were summarized by affiliation type. Unadjusted 3-year survival was evaluated using Kaplan-Meier curves, as well as 5-year survival as a sensitivity analysis. Quality metrics were compared across affiliate status using χ^2^ and Wilcoxon tests.

To assess differences in 90-day mortality between affiliates and top-ranked hospitals, we estimated a series of random effects logistic regression models. A separate model was estimated for each procedure and 1 for all procedures pooled. Effects are reported as odds ratios (ORs) representing risk of 90-day mortality for patients at affiliate hospitals.

To assess differences in long-term survival, we estimated time-to-event models for each cancer type and all cancers pooled. Schoenfeld residual tests rejected the proportional hazards assumptions for all cancer types and all types pooled.^25^ Therefore, we evaluated parametric time-to-event models using Akaike Information Criteria, identifying the γ distribution as providing the best fit.^26^ Mixed-effects γ models for each cancer type included the same risk adjusters as for 90-day mortality, with additional indicators in the lung, colon, and gastric models for procedure type. The pooled model also included indicators for cancer type. Time ratios (TRs) were reported to represent relative total survival time for patients at affiliate hospitals compared with those at top-ranked hospitals.

To account for clustering effect, all models included nested random intercepts that were allowed to vary across hospitals. Variance decomposition analysis indicated that there was no additional correlation within networks. To account for potential selection bias, we estimated propensity models in which the outcome was treatment at an affiliate hospital. A separate model was estimated for each procedure or cancer type and used to estimate a propensity score; these propensity scores were incorporated as covariates in all adjusted models (eAppendix in the Supplement).

Missing variables, excluding pathological stage, were imputed using multiple imputation (eTable 1 in the Supplement); because of the prognostic significance of stage, patients with incomplete pathological stage data (7359 patients [6.1%]) were excluded from all adjusted models (eTable 2 in the Supplement). However, a sensitivity analysis that included patients with missing pathological stage in the 90-day mortality models did not affect results.

Statistical analyses were performed using SAS statistical software version 9.4 (SAS Institute) and Stata statistical software version 16 (StataCorp). *P* values were 2-sided, and statistical significance was set at .05. Data were analyzed from July through December 2019.

Several sensitivity analyses were performed. Annual surgical volume for each procedure at each hospital was incorporated into adjusted models. We analyzed 30-day, instead of 90-day, mortality. We performed our analysis excluding the 3 largest networks. We also performed the analysis restricting to networks in which both top-ranked hospitals and their specific affiliates were represented. We further stratified models by age (<65 year vs ≥65 years). A further analysis was performed including administration of adjuvant chemotherapy in patients with gastric cancer patients, which is not a COC quality measure.

## Results

### Patients and Hospitals

A total of 56 top-ranked hospitals and 206 affiliates were included in analysis (eFigure 1 in the Supplement). The median (range) number of affiliates for each top-ranked hospital was 3 (0-29). Median (interquartile range [IQR]) follow-up was 629 (327-1129) days at top-ranked hospitals and 539 (269-974) days at affiliates. Overall, 119 834 patients underwent complex cancer treatment within top-ranked networks, including 79 981 patients (66.7%) at top-ranked cancer hospitals (median [IQR] age, 66 [58-74] years; 40 910 [54.9%] men) and 39 853 patients (33.3%) at brand-sharing affiliates of top-ranked hospitals (median [IQR] age, 69 [60-77] years; 19 004 [50.0%] men). The distribution of surgical procedures was heterogenous across hospital types, as affiliates performed 21 354 of 40 220 partial colectomies (53.1%), but 1392 of 11 161 Whipple procedures (12.5%) of (Table). The proportion of surgical procedures performed by affiliates within top-ranked networks increased over time, from 24.6% in 2012 to 40.2% in 2016.

**Table.** Patient, Tumor, and Treatment Characteristics Stratified by Hospital Type

Characteristic	Patients, No. (%)^a^	
	Top-ranked hospitals (n = 74 466)	Affiliate hospitals (n = 38 009)
Age, median (IQR), y^b^	66 (58-74)	69 (60-77)
Men	40 910 (54.9)	19 004 (50.0)
Race		
White	62 530 (84.0)	32 458 (85.4)
Black	6872 (9.2)	3810 (10.0)
Other or missing	5064 (6.8)	1741 (4.6)
Procedure type^c^		
Esophagectomy	3801 (5.1)	415 (1.1)
Partial gastrectomy	4513 (6.1)	1289 (3.4)
Total gastrectomy	2013 (2.7)	440 (1.2)
Total colectomy	1489 (2.0)	694 (1.8)
Partial colectomy	18 686 (25.1)	21 354 (56.2)
Lobectomy	21 563 (29.0)	9276 (24.4)
Pneumonectomy	1417 (1.9)	381 (1.0)
Proctectomy	3168 (4.2)	1333 (3.5)
Whipple	9769 (13.1)	1392 (3.7)
Cystectomy	8047 (10.8)	1435 (3.8)
Charlson-Deyo comorbidity scale score		
0	48 497 (65.1)	24 144 (63.5)
1	18 234 (24.5)	9103 (23.9)
2	5294 (7.1)	3057 (8.0)
≥3	2441 (3.3)	1705 (4.5)
Distance to hospital, median (IQR), miles^d^	26.1 (9.6-66.3)	8.2 (3.8-18.6)
Hospital type		
Academic/National Cancer Institute	72 458 (97.3)	7612 (20.0)
Comprehensive community	0	22 985 (60.5)
Community	0	3766 (9.9)
Integrated network program	2008 (2.7)	3646 (9.6)
Mean annual surgical volume, median (IQR)^e^	70.8 (39.2-116.8)	32.0 (15.8-48.0)
Diagnostic or staging procedure performed, %	59.5	60.9
Pathological stage group^f^		
0	2531 (3.4)	1024 (2.7)
I	25 588 (34.4)	13 118 (34.5)
II	25 928 (34.8)	12 109 (31.9)
III	16 901 (22.7)	10 384 (27.3)
IV	3518 (4.7)	1374 (3.6)
Tumor grade		
Well differentiated	7774 (10.4)	4819 (12.7)
Moderately differentiated	31 575 (42.4)	20 432 (53.8)
Poorly differentiated	20 159 (27.1)	8328 (21.9)
Undifferentiated	5805 (7.8)	1728 (4.5)
Missing	9153 (12.3)	2702 (7.1)
Chemotherapy		
No chemotherapy	38 884 (52.2)	24 022 (63.2)
Neoadjuvant chemotherapy	14 855 (20.0)	2665 (7.0)
Adjuvant chemotherapy	16 813 (22.6)	10 007 (26.3)
Missing	3914 (5.3)	1315 (3.5)
Radiation		
No radiation	62 309 (83.7)	34 792 (91.5)
Neoadjuvant radiation	8099 (10.9)	1646 (4.3)
Adjuvant radiation	3501 (4.7)	1437 (3.8)
Missing	557 (0.7)	134 (0.4)
Unplanned 30-d readmission^g^	3782 (5.1)	1915 (5.0)
Negative surgical margins	68 647 (92.2)	35 472 (93.3)
Time from diagnosis to surgical treatment, median (IQR), d^h^	30 (10-53)	17 (0-37)
Minimally invasive approach	31 066 (41.7)	17 295 (45.5)
Adherence to COC quality measures, % (95% CI)^i^		
Colon cancer		
Scenario-specific use of adjuvant chemotherapy	89.0 (88.1-89.8)	89.9 (89.1-90.6)
≥12 Regional lymph nodes	95.1 (94.8-95.5)	91.5 (91.1-91.8)
Lung cancer		
Scenario-specific use of adjuvant chemotherapy	92.5 (91.7-93.2)	93.2 (92.2-94.2)
≥10 Lymph nodes	50.7 (50.0-51.4)	45.6 (44.7-46.6)
Chemotherapy use around gastrectomy, % (95% CI)^j^	42.6 (40.9-44.3)	41.8 (37.9-45.8)

Abbreviations: COC, Commission on Cancer; IQR, interquartile range.

^a^ Total percentages for many variables will not yield 100%, because not all categories are shown (eg, unknown, indeterminate). The values exclude patients missing pathologic stage (to represent the population in the adjusted analyses) for all variables except procedural volume, and quality measures, because volume relationships with other outcomes would not be dependent on pathologic stage, and bias could be created in quality measures by excluding patients missing pathologic stage.

^b^ Age is shown as a continuous variable. However, in adjusted models, age was considered as a categorical variable because risk was felt to be heterogenous across age (ie, elderly patients were considered higher risk).

^c^ The percentages in the parentheses represent the distribution of each level for a particular variable among the hospital cohort.

^d^ Distance was calculated as the most direct distance between the patient’s and hospital’s zip codes.

^e^ Median annual volume calculated for all procedures included in the analysis, including patients with missing 90-day mortality.

^f^ Some patients received induction therapy, so they were classified as pathologic stage 0.

^g^ Restricted to patients who traveled less than 50 miles for cancer treatment.

^h^ Restricted to patients for whom surgical treatment was the first treatment.

^i^ Proportion of patients who were adherent with COC measure within each cohort.

^j^ Includes patients who met National Cancer Care Network guidelines for chemotherapy around gastrectomy for adenocarcinoma (ie, *t*>2, N>1, or M>0). Excludes patients older than 80 years.

Among the modest differences in patient sociodemographic characteristics and health across hospital cohorts, a greater proportion of patients at affiliate hospitals were 75 years or older (12 817 patients [32.2%] vs 17 993 patients [22.5%]; *P* < .001) (eTable 1 in the Supplement). Patients at top-ranked cancer hospitals were more likely to receive preoperative chemotherapy (14 855 patients [20.8%] vs 2655 patients [7.8%]; *P* < .001) and preoperative radiation (8099 patients [12.3%] vs 1646 patients [5.1%]; *P* < .001) and to have traveled farther distances between home and hospital (median [IQR] distance, 26.1 [9.6-66.3] miles vs 8.2 [3.8-18.6] miles; *P* < .001). A staging or diagnostic procedure was performed in 60.9% of patients at top-ranked hospitals and 61.5% of patients at affiliates.

Top-ranked hospitals tended to have higher median (IQR) annual procedural volumes (68.2 [37.8-111.4] procedures) compared with affiliates (31.8 [15.0-47.6] procedures) (*P* < .001) and the differential varied by procedure (eTable 2 in the Supplement). Additionally, a greater proportion of top-ranked hospitals than affiliates were academic hospitals (72 458 hospitals [97.3%] vs 7612 hospitals [20.0%]; *P* < .001). Additional patient and hospital characteristics are described in eTable 1 in the Supplement.

### Surgical Quality Measures

Several metrics for quality were compared across hospital types (Table). Complete removal of the tumor (ie, negative surgical margins) was slightly less common among patients at top-ranked hospitals than among those at affiliates (68 647 patients [92.3%] vs 35 472 patients [93.3%]; *P* < .001). Furthermore, the interval between diagnosis and surgical treatment was longer for patients at the top-ranked cancer hospitals than those at affiliates (median [IQR], 30 [10-54] days vs 17 [0-37] days; *P* < .001) and varied by procedure (eTable 3 in the Supplement).

Adherence with COC quality measures was assessed for patients with lung or colon cancer, as they were cared for by the largest number of hospitals. Affiliates and top-ranked hospitals were similarly adherent with the adjuvant chemotherapy measures. However, top-ranked hospitals were more likely than affiliate hospitals to analyze the recommended number of lymph nodes for colon cancer (95.1% [95% CI, 94.8%-95.5%] adherence vs 91.5% [95% CI, 91.1%-91.8%] adherence; *P* < .001) and lung cancer (50.7% [95% CI, 50.0%-51.4%] adherence vs 45.6% [95% CI, 44.7%-46.6%] adherence; *P* < .001) (Table). Adherence with recommended chemotherapy for gastric cancer, which is not a COC quality measure, was assessed as a sensitivity analysis (Table). Adherence with recommended chemotherapy after gastrectomy was similar in top-ranked hospitals (42.6% [95% CI, 40.9%-44.3%] adherence) and affiliates (41.8% [95% CI, 37.9%-45.8%] adherence) (*P* = .72), but both were considerably less adherent than what was observed for the COC chemotherapy quality measures.

### Readmission and Short-term Survival

**Figure 1.**
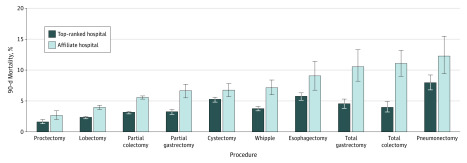
Observed 90-Day Mortality Rates Across Procedures Whiskers indicate 95% CI.

**Figure 2.**
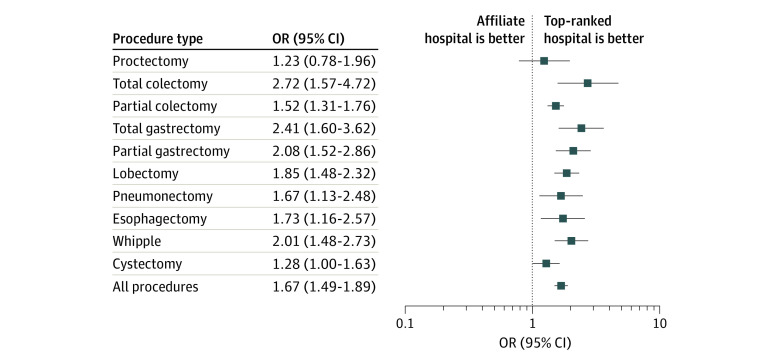
Adjusted 90-Day Mortality Across Surgical Procedures Logistic regression results for each procedure were calculated with top-ranked hospitals serving as the reference. The models were adjusted for year, age category, insurance type, Charlson-Deyo group, income category, sex, pathological stage, grade, receipt of preoperative chemotherapy (within 180 days of surgical treatment), receipt of preoperative radiation therapy (within 180 days of surgical treatment), and, for relevant procedures, whether they were extended or not. Boxes indicate odds ratio (OR), and whiskers, 95% CI.

Overall, unplanned postoperative 30-day readmission rates were similar across hospital types (Table; eTable 4 in the Supplement). The unadjusted 90-day mortality rates were statistically significantly higher at the affiliates across all procedures (Figure 1). The adjusted 90-day mortality risk for all procedures combined was statistically significantly higher at the affiliates compared with the top-ranked cancer hospitals (OR, 1.67 [95% CI, 1.49-1.89]; *P* < .001) (Figure 2). The adjusted 90-day mortality risk varied by procedure from proctectomy (OR, 1.23 [95% CI, 0.78-1.96]; *P* = .38), to total colectomy (OR, 2.72 [95% CI, 1.57-4.72]; *P* < .001).

### Long-term Survival

**Figure 3.**
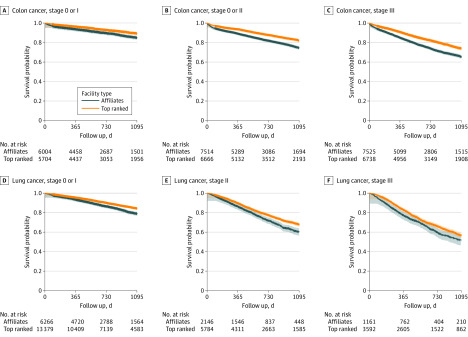
Stage-Stratified Unadjusted 3-Year Survival for Surgically Managed Colon and Lung Cancer Crosses indicate censored patients.

Unadjusted 3-year survival was significantly superior at top-ranked hospitals for each cancer type (eFigure 2 in the Supplement). Stage-stratified unadjusted survival demonstrated significantly worse survival at affiliates than at top-ranked hospitals for lung and colon cancer (Figure 3).

**Figure 4.**
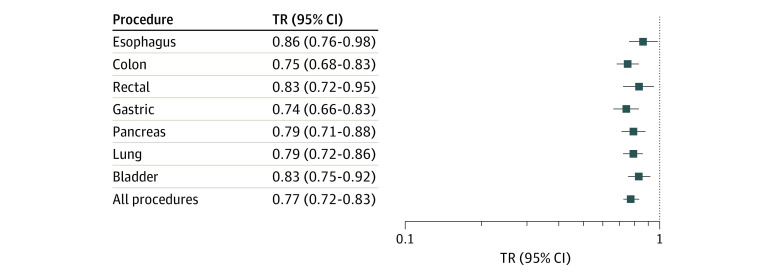
Survivorship at Affiliate Hospitals Compared With Top-Ranked Hospitals The models were adjusted for year, age category, insurance type, Charlson-Deyo group, income category, sex, pathological stage, grade, receipt and timing of chemotherapy, receipt and timing of radiation therapy, sequence, and, for relevant procedures, whether they involved extended resections or not. Boxes indicate time ratio (TR), and whiskers, 95% CI. A total of 886 patients (0.7%) did not have a precise date of diagnosis and were excluded from survival results.

In adjusted analyses using parametric survival models, patients treated at affiliate hospitals experienced statistically significantly less survival time compared with patients treated at top-ranked hospitals (TR, 0.77 [95% CI, 0.72-0.83]; *P* < .001) (Figure 4). The magnitude of survival reduction for affiliates varied across cancers but was significantly lower for all cancers. A sensitivity analysis that excluded mortality within 90 days of surgical treatment (ie, only included patients who survived beyond the perioperative period) also demonstrated lower survival at affiliates (pooled TR, 0.87 [95% CI, 0.82-0.92]; *P* < .001) (eFigure 3 in the Supplement).

### Sensitivity Analyses

Adjusting for annual procedure volume did not eliminate survivorship differences at affiliates vs top-ranked hospitals in terms of short-term (90-day mortality risk at affiliates: OR, 1.35 [95% CI, 1.15-1.58]; *P* < .001) or long-term (TR, 0.82 [95% CI, 0.74-0.90]; *P* < .001) survivorship (eFigure 4 and eFigure 5 in the Supplement). Additional sensitivity analyses restricting hospital subsets or stratifying by age (eFigure 3 in the Supplement) did not change the direction of the results.

## Discussion

This cohort study found that short- and long-term survivals after complex cancer treatment were superior at top-ranked cancer hospitals compared with brand-sharing affiliates. The nearly 1.7-fold difference in probability of 90-day mortality risk at affiliates mirrors a 2019 report by Hoag et al^13^ within the Medicare population. Our study extends these short-term survival findings in several important ways. First, our cohort is 4-fold larger (approximately 120 000 patients vs 29 000 in the study by Hoag et al^13^). Second, all adults were considered, whereas the study by Hoag et al was limited to adults older than 65 years. Third, our adjusted models were enhanced by the inclusion of more tumor attributes (eg, stage, grade) and treatment data (eg, chemotherapy, radiation). Overall, our findings may serve to fortify concerns that considerable differences in perioperative safety may exist between top-ranked hospitals and affiliates.

We found that long-term outcomes were superior at top-ranked hospitals for each cancer type studied. Importantly, the survival advantage was observed for colon cancer, which is commonly managed in the community (ie, affiliates performed more partial colectomies than the top-ranked hospitals). The differences in long-term survival were not entirely explained by advantages in surgical mortality, given survival at top-ranked hospitals was superior even when deaths occurring in the perioperative period were excluded (ie, landmarked at 90 days). This suggests differences in care may include nonsurgical as well as surgical components.

The top-ranked hospitals performed more than 2-fold the annual volume of affiliate hospitals, which is potentially important because higher surgical volumes have been associated with advantages in surgical mortality,^27,28^ and long-term survival.^29,30,31^ However, sensitivity analyses demonstrated that differences in surgical volume did not fully explain the differences in survival. That is, while volume adjustment did mitigate a portion of the differential (ie, narrowed margin between affiliates and top-ranked hospitals, with some procedures losing significance), the overall finding of inferior survival at affiliates remained. This is consistent with our findings when volume was included in our Medicare database study.^13^ It should be noted that our study was not designed to explain the etiological origin of the differential, simply to assess the magnitude of the differential. We recognize that for some procedures in some networks, the optimal survival may require lower volume hospitals to stop performing certain operations.

A number of quality metrics known to affect long-term survival (eg, surgical margin status,^32^ compliance with COC chemotherapy and lymph node measures^17^) were similar between hospital cohorts. The interval of time between diagnosis and surgical treatment, which is potentially associated with survival,^33,34,35^ favored the affiliate hospitals (ie, opposite direction of observed survivorship advantage). This may reflect a greater travel distance for patients being cared for at top-ranked hospitals or workflow congestion from treating higher patient volumes at top-ranked hospitals. If affiliates performed more emergency surgical procedures (generally limited to colon or gastric cancers), this could decrease the time to treatment at affiliates and could also negatively affect survivorship (ie, obstructing and perforated cancers typically are more advanced). While the NCDB does not capture emergent surgical treatment status, emergent treatments were excluded from our Medicare study, and affiliate patients remained 1.4-fold as likely to die within 90 days of surgical treatment.^13^

We hypothesized that affiliated hospitals would have worse outcomes for patients undergoing complex cancer treatment. There may be multiple mechanisms to explain outcome differences (eg, physician training, hospital resources, degree of network integration, and surgical volumes). By demonstrating that brand-sharing does not equate to quality sharing, we hope to motivate efforts to leverage unique levels of connectivity and collaboration provided by networks. The onus is now on the medical community to analyze these survival differences to identify opportunities to improve care across the networks.

### Limitations

There are several limitations to our study. In addition to the limitations typical of observational studies, selection bias may exist, as patients chose hospitals, and this choice may be associated with patient factors that are also associated with their outcomes. Although we addressed this by incorporating propensity score adjustment in our models, there may be confounders that were not available (eg, marital status). In addition, the study was limited to patients undergoing surgical treatment at COC-accredited hospitals, and COC accreditation has been associated with better outcomes.^36^ Therefore, we may be studying the better affiliates, which may minimize the differential. Another limitation is that cause of death and date of recurrence data are not available. A proportion of patients likely died of noncancer causes, unrelated to their surgical or oncological care (eg, diabetes management, nutrition), and it is plausible that differences in noncancer causes drive the differences in overall mortality. On the other hand, the unadjusted survival focused in the first 3 years after cancer treatment, a period in which the risk of cancer recurrence would be at its highest. Related to this, the completeness of the staging evaluation is unknown. It is possible that top-ranked hospitals performed more accurate staging evaluations, which could favorably affect the survival in the top-ranked cohort. Since NCDB does not capture use of imaging, we examined the use of staging and diagnostic procedures as a surrogate for the staging workup and found the frequency to be similar between top-ranked hospitals (60.9%) and affiliates (61.5%). Additionally, the nature of the affiliation relationship (ie, limited vs extensive integration) or duration is unknown. In our previous study of top-ranked hospitals networks,^13^ the median (IQR) duration of affiliation by end of study was 51 (27-137) months. That being said, patients are unlikely to have a comprehensive understanding of the nature of an affiliation relationship when they are potentially influenced by brand-sharing.

## Conclusions

These findings suggest that safety and potentially the effectiveness of the overall care of patients undergoing complex cancer treatment appears to be superior at top-ranked cancer hospitals compared with affiliate hospitals. Additional efforts to characterize survival within networks around top-ranked cancer hospitals could identify collaborative approaches to improve care and inform patient choice for hospitals.

## Notes

Supplement. **eFigure 1.** Consort Diagram **eFigure 2.** Unadjusted 3-Year Survival Curves From Kaplan Meier Analyses for Each Cancer Type With All Stages Combined **eFigure 3.** Survivorship at Affiliate Hospitals Compared With Top-Ranked Hospitals Landmarked at 90 Days **eFigure 4.** Adjusted 90-Day Mortality Across Surgical Procedures Adjusting For Annual Procedure Volume **eFigure 5.** Survivorship at Affiliate Hospitals Compared With Top-Ranked Hospitals, Adjusting for Annual Procedural Volume **eTable 1.** Patient, Tumor, and Treatment Characteristics by Hospital Type **eTable 2.** Annual Procedure Volumes **eTable 3.** Time to Treat Across Different Procedures **eTable 4.** Unplanned Readmission Within 30 Days **eAppendix.** Supplemental Methods
