# Supplementary material for: Survival After Cancer Treatment at Top-Ranked US Cancer Hospitals vs Affiliates of Top-Ranked Cancer Hospitals
Source: JAMA Netw Open. 2020 May 26;3(5):e203942. doi: 10.1001/jamanetworkopen.2020.3942 (PMC7251445; doi:10.1001/jamanetworkopen.2020.3942)
Supplement: Supplement. — eFigure 1. Consort Diagram eFigure 2. Unadjusted 3-Year Survival Curves From Kaplan Meier Analyses for Each Cancer Type With All Stages Combined eFigure 3. Survivorship at Affiliate Hospitals Compared With Top-Ranked Hospitals Landmarked at 90 Days eFigure 4. Adjusted 90-Day Mortality Across Surgical Procedures Adjusting For Annual Procedure Volume eFigure 5. Survivorship at Affiliate Hospitals Compared With Top-Ranked Hospitals, Adjusting for Annual Procedural Volume eTable 1. Patient, Tumor, and Treatment Characteristics by Hospital Type eTable 2. Annual Procedure Volumes eTable 3. Time to Treat Across Different Procedures eTable 4. Unplanned Readmission Within 30 Days eAppendix. Supplemental Methods [file jamanetwopen-3-e203942-s001.pdf]

Supplementary Online Content

Boffa DJ, Mallin K, Herrin J, et al. Survival after cancer treatment at top-ranked US cancer hospitals vs affiliates of top-ranked cancer hospitals. *JAMA Netw Open*. 2020;3(5):e203942. doi:10.1001/jamanetworkopen.2020.3942

eFigure 1. Consort Diagram

eFigure 2. Unadjusted 3-Year Survival Curves From Kaplan Meier Analyses for Each Cancer Type With All Stages Combined

eFigure 3. Survivorship at Affiliate Hospitals Compared With Top-Ranked Hospitals Landmarked at 90 Days

eFigure 4. Adjusted 90-Day Mortality Across Surgical Procedures Adjusting For Annual Procedure Volume

eFigure 5. Survivorship at Affiliate Hospitals Compared With Top-Ranked Hospitals, Adjusting for Annual Procedural Volume

eTable 1. Patient, Tumor, and Treatment Characteristics by Hospital Type

eTable 2. Annual Procedure Volumes

eTable 3. Time to Treat Across Different Procedures

eTable 4. Unplanned Readmission Within 30 Days

eAppendix. Supplemental Methods

This supplementary material has been provided by the authors to give readers additional information about their work.

© 2020 Boffa DJ et al. *JAMA Network Open.*

eFigure 1. Consort Diagram

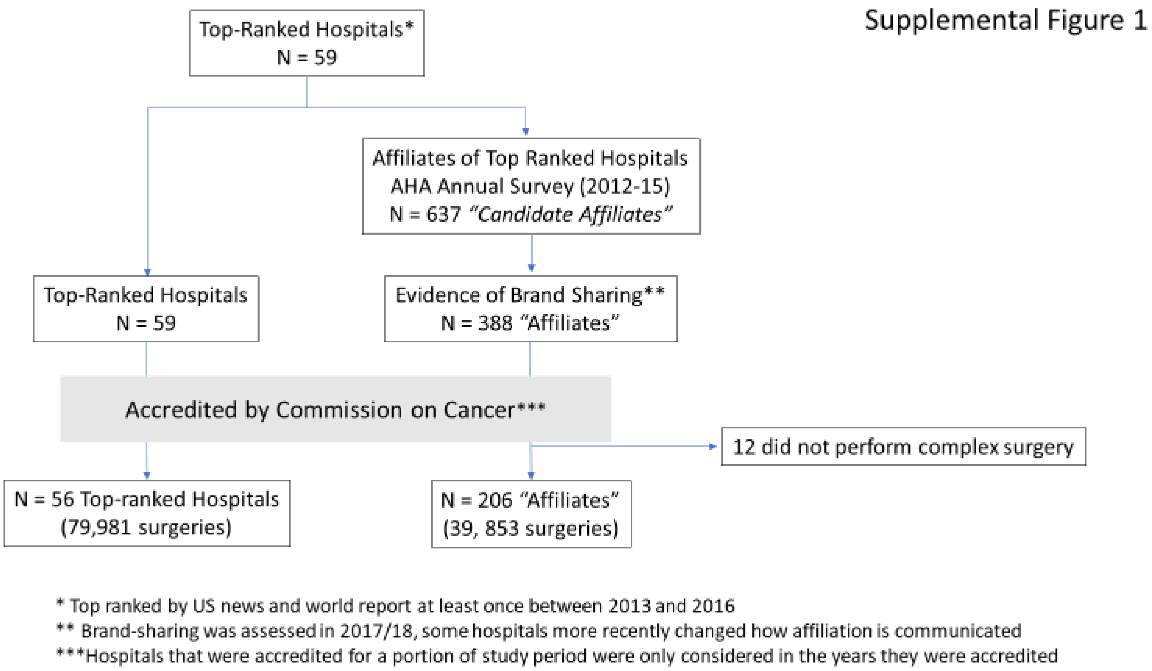

© 2020 Boffa DJ et al. *JAMA Network Open.*

eFigure 2. Unadjusted 3-Year Survival Curves From Kaplan Meier Analyses for Each Cancer Type With All Stages Combined

Supplemental Figure 2

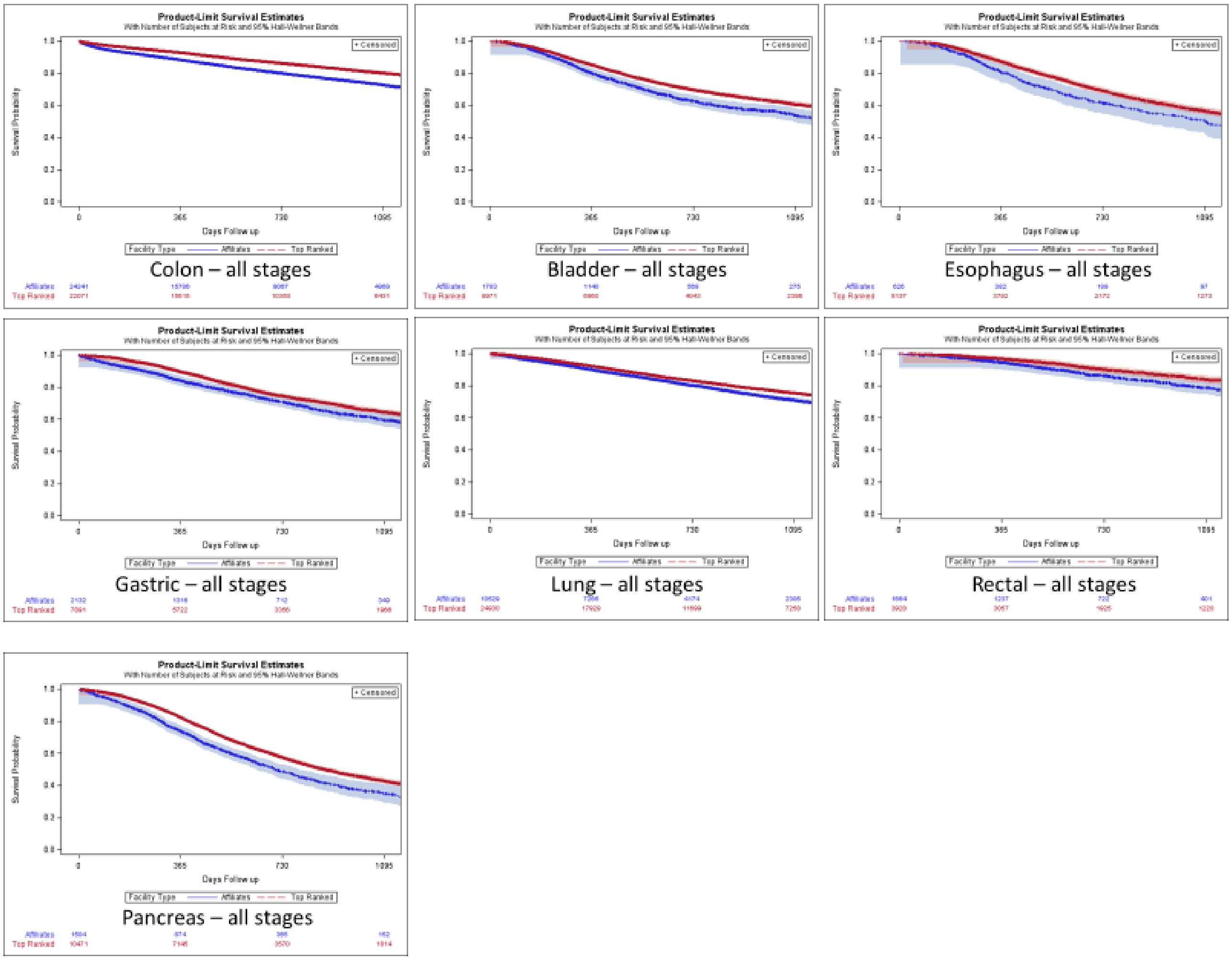

eFigure 2: Survival at top-ranked hospitals is shown in red, and survival at affiliates is shown in blue. 3-year survival was felt to be best supported by the median follow up, but 5-year survival is also provided in Supplemental Table 2.

© 2020 Boffa DJ et al. *JAMA Network Open.*

eFigure 3. Survivorship at Affiliate Hospitals Compared With Top-Ranked Hospitals Landmarked at 90 Days

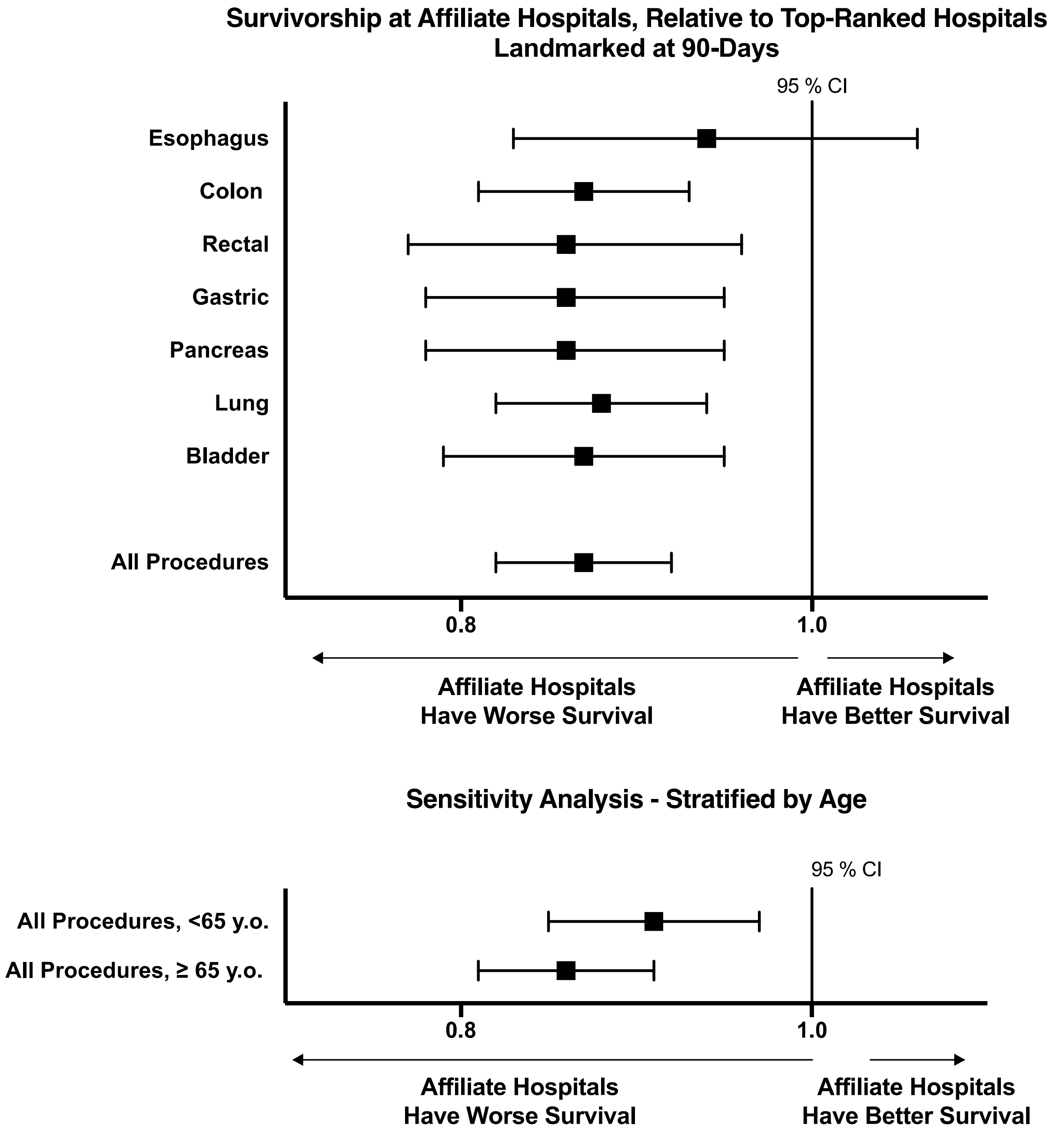

Figure 3: A forest plot of gamma model results for each procedure is shown, with survivorship at top ranked hospitals serving as reference. The models were adjusted for year, age category, insurance type, Charlson-Deyo group, income category, sex, pathologic stage, grade, receipt and timing of chemotherapy, receipt and timing of radiation therapy, sequence, and, for relevant procedures, whether they involved "extended" resections or not. The "box"

© 2020 Boffa DJ et al. *JAMA Network Open.*

represents the Time Ratio (TR), while the “whiskers” represent the 95% confidence intervals. The light gray at the bottom indicates a sensitivity analysis of patients stratified by age.

© 2020 Boffa DJ et al. *JAMA Network Open.*

eFigure 4. Adjusted 90-Day Mortality Across Surgical Procedures Adjusting For Annual Procedure Volume

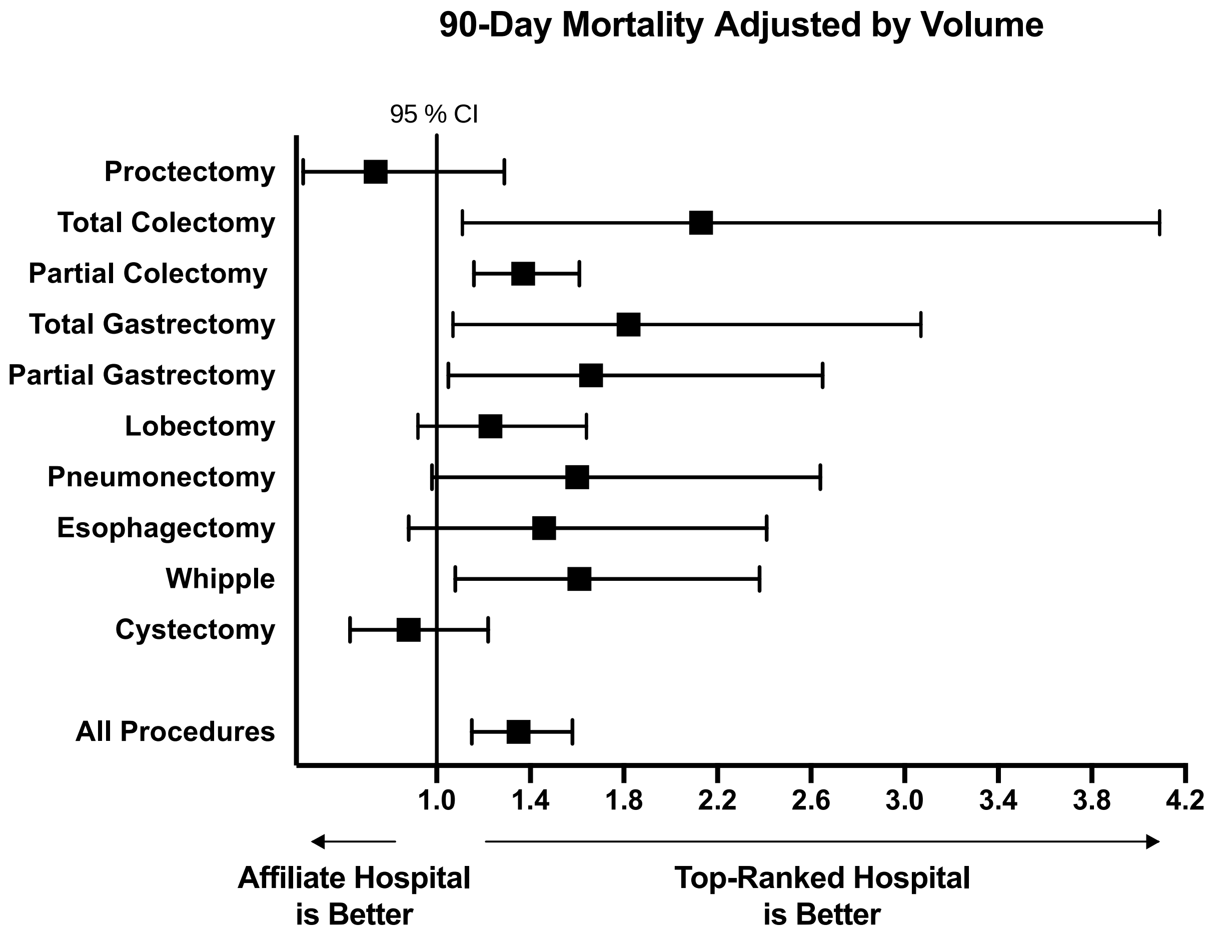

Figure 4: A forest plot of logistic regression results for each procedure are shown, with top-ranked hospitals serving as the reference. Mortality was significantly lower at top-ranked hospitals for all procedures excepted proctectomy and cystectomy. The "box" represents the odds ratio, while the "whiskers" represent 95% confidence intervals. The models were adjusted for year, age category, insurance type, Charlson-Deyo group, income category, sex, pathologic stage, grade, receipt of preoperative chemotherapy (within 180 days of surgery), receipt of preoperative radiation therapy (within 180 days of surgery), and, for relevant procedures, whether they were extended or not. In addition, the annual volume for each procedure at each hospital was included in the adjustment.

© 2020 Boffa DJ et al. *JAMA Network Open.*

eFigure 5. Survivorship at Affiliate Hospitals Compared With Top-Ranked Hospitals, Adjusting for Annual Procedural Volume

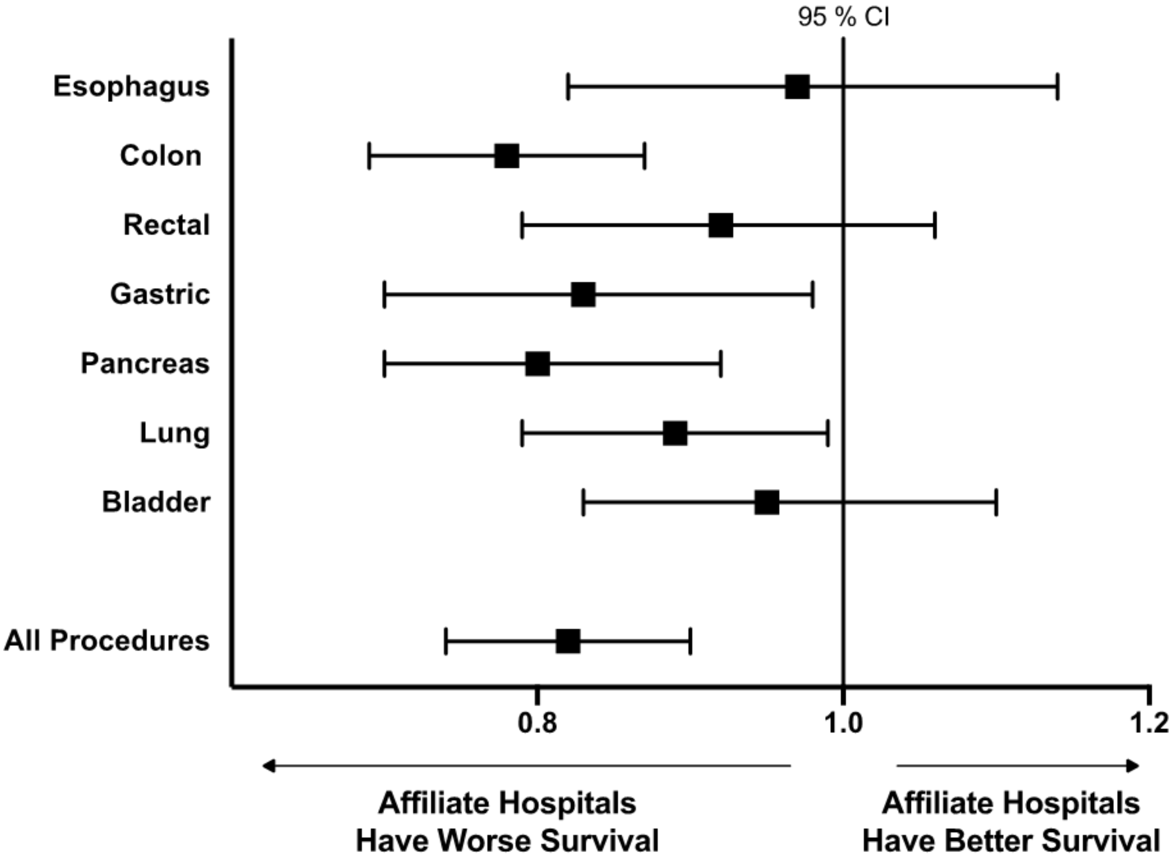

Figure 5: A forest plot of gamma model results for each procedure is shown, with survivorship at top ranked hospitals serving as reference. The models were adjusted for year, age category, insurance type, Charlson-Deyo group, income category, sex, pathologic stage, grade, receipt and timing of chemotherapy, receipt and timing of radiation therapy, sequence, and, for relevant procedures, whether they involved “extended” resections or not. In addition, the annual volume for each procedure at each hospital was included in the adjustment. The “box” represents the Time Ratio (TR), while the “whiskers” represent the 95% confidence intervals. The light gray at the bottom indicates a sensitivity analysis of patients stratified by age.

© 2020 Boffa DJ et al. *JAMA Network Open.*

eTable 1. Patient, Tumor, and Treatment Characteristics by Hospital Type

	Hospital Type	
	**Top-Ranked (n=79,981 patients)**	**Affiliate (n=39,853 patients)**
**Cancer type (%)**		
Esophagus	4,899 (6.1)	564 (1.4)
Gastric	7,465 (9.3)	1,985 (5.0)
Colon	20,830 (26.0)	22,689 (56.9)
Rectal	3,632 (4.5)	1,565 (3.9)
Pancreas	10,088 (12.6)	1,428 (3.6)
Lung	23,638 (29.6)	9,923 (24.9)
Bladder	9,429 (11.8)	1,699 (4.3)
**Procedure Type**		
Esophagectomy	4,899 (6.1)	564 (1.4)
Partial Gastrectomy	5,239 (6.5)	1,510 (3.8)
Total Gastrectomy	2,226 (2.8)	475 (1.2)
Total Colectomy	1,547 (1.9)	720 (1.8)
Partial Colectomy	19,283 (24.1)	21,969 (55.1)
Lobectomy	22,147 (27.7)	9,528 (23.9)
Pneumonectomy	1,491 (1.9)	395 (1.0)
Proctectomy	3,632 (4.5)	1,565 (3.9)
Whipple	10,088 (12.6)	1,428 (3.6)
Cystectomy	9,429 (11.8)	1,699 (4.3)
**Diagnosis Year**		
2012	15,413 (19.3)	5,031 (12.6)
2013	16,219 (20.3)	6,379 (16.0)
2014	16,662 (20.8)	8,184 (20.5)
2015	16,585 (20.7)	10,108 (25.4)
2016	15,102 (18.9)	10,151 (25.5)
**Age in years**		
Median (IQR)	66 (58-74)	69 (60-77)
18-44	4,136 (5.2)	1,551 (3.9)
45-54	9,971 (12.5)	4,299 (10.8)
55-64	20,947 (26.2)	8,723 (21.9)
65-74	26,934 (33.7)	12,463 (31.3)
75-84	15,156 (18.9)	9,564 (24.0)
>=85	2,837 (3.5)	3,253 (8.2)
**Sex**		
Male	44,558 (55.7)	20,078 (50.4)
Female	35,423 (44.3)	19,775 (49.6)
**Race**		
White	67,329 (84.2)	34,059 (85.5)
Black	7,297 (9.1)	3,983 (10.0)
Other, Missing	5,355 (6.7)	1,811 (4.5)
**Charlson Deyo Score**		

© 2020 Boffa DJ et al. *JAMA Network Open.*

0	52,442 (65.6)	25,436 (63.8)
1	19,377 (24.2)	9,477 (23.8)
2	5,589 (7.0)	3,177 (8.0)
>=3	2,573 (3.2)	1,763 (4.4)
**Insurance**		
No insurance	1,272 (1.6)	749 (1.9)
Private insurance	31,051 (38.8)	12,290 (30.8)
Medicaid	4,345 (5.4)	2,282 (5.7)
Medicare	41,031 (51.3)	23,698 (59.5)
Other Government	994 (1.2)	347 (0.9)
Missing	1,288 (1.6)	487 (1.2)
**Median income quintiles[2]**		
Lowest 0- 20%	9,157 (11.4)	4,647 (11.7)
21-40%	12,796 (16.0)	6,292 (15.8)
41-60%	13,420 (16.8)	7,386 (18.5)
61-80%	15,355 (19.2)	8,511 (21.4)
Highest 81-100%	28,023 (35.0)	12,570 (31.5)
Missing	1,230 (1.5)	447 (1.1)
**Urban rural designation of patient county[3]**		
Metro area >= 1 million	45,628 (57.0)	23,151 (58.1)
Metro area 250,000-1,000,000	13,201 (16.5)	6,251 (15.7)
Metro area < 250,000	6,776 (8.5)	4,308 (10.8)
Non-metro area < 2,500 adjacent to metro area	560 (0.7)	188 (0.5)
Non-Metro area <2,500 not adjacent to metro area	651 (0.8)	342 (0.9)
Non-metro 2,500-19,999 adjacent to metro area	3,416 (4.3)	1,854 (4.6)
Non-metro 2,500-19,999 not adjacent to metro area	2,004 (2.5)	685 (1.7)
Non-metro >=20,000 adjacent to metro area	4,459 (5.6)	1,915 (4.8)
Non-metro >= 20,000 not adjacent to metro area	1,038 (1.3)	424 (1.1)
Missing	2,248 (2.8)	735 (1.8)
**Distance to hospital[4]**		
Median (interquartile range)	26.7 (9.8-67.3)	8.3 (3.8-18.7)
Missing	380 (.5)	78 (0.2)

© 2020 Boffa DJ et al. *JAMA Network Open.*

< 10 miles	20,067 (25.1)	22,482 (56.4)
10-24 miles	18,289 (22.9)	10,158 (25.5)
25-49 miles	14,782 (18.5)	4,300 (10.8)
50-74 miles	8,594 (10.7)	1,357 (3.4)
75-99 miles	4,916 (6.1)	564 (1.4)
100-199 miles	7,699 (9.6)	547 (1.4)
>=200 miles	5,254 (6.6)	367 (0.9)
**Median Days between diagnosis and surgery (IQR)[5]**	30.0 (10-54)	17.0 (0-37)
**Clinical Stage Group[6]**		
Missing	21,584 (27.0)	18,171 (45.6)
0	1,453 (1.8)	799 (2.0)
I	26,496 (33.1)	10,931 (27.4)
II	19,417 (24.3)	6,341 (15.9)
III	11,031 (13.8)	3,611 (9.1)
**Clinical Stage Group Excluding Colon**		
Missing	8,388 (14.2)	2,919 (17.0)
0	739 (1.2)	179 (1.0)
I	22,862 (38.6)	7,983 (46.5)
II	17,465 (29.6)	3,941 (23.0)
III	9,697 (16.4)	2,142 (12.5)
**Pathologic Stage Group**		
Missing	5,515 (6.9)	1,844 (4.6)
0	2,531 (3.2)	1,024 (2.6)
I	25,588 (32.0)	13,118 (32.9)
II	25,928 (32.4)	12,109 (30.4)
III	16,901 (21.1)	10,384 (26.1)
IV	3,518 (4.4)	1,374 (3.4)
**Tumor Grade**		
Well differentiated	8,240 (10.3)	5,106 (12.8)
Moderately differentiated	32,990 (41.2)	21,035 (52.8)
Poorly differentiated	21,496 (26.9)	8,666 (21.7)
Undifferentiated	6,709 (8.4)	1,895 (4.7)
Missing	10,546 (13.2)	3,151 (7.9)
**Cancer Sequence**		
First or only primary	61,982 (77.5)	31,548 (79.2)
2nd or higher primary	17,999 (22.5)	8,305 (20.8)
**Hospital Census Division[7]**		
New England	5,060 (6.3)	3,433 (8.6)
Middle Atlantic	21,268 (26.6)	7,935 (19.9)
South Atlantic	17,019 (21.3)	9,577 (24.0)
East North Central	15,492 (19.4)	9,007 (22.6)

© 2020 Boffa DJ et al. *JAMA Network Open.*

East South Central	1,771 (2.2)	934 (2.3)
West North Central	8,325 (10.4)	3,528 (8.8)
West South Central	1,897 (2.4)	1,480 (3.7)
Mountain	2,289 (2.9)	2,328 (5.8)
Pacific	6,860 (8.6)	1,631 (4.1)
**Commission on Cancer Hospital Category Type[8]**		
Academic/NCI	77,850 (97.3)	7,972 (20.0)
Comprehensive Community	0	24,165 (60.6)
Community	0	3,918 (9.8)
Integrated Network Program	2,131 (2.7)	3,798 (9.5)
**Average Annual Surgical Volume[9]**		
Median (IQR)	68.2 (37.8-111.4)	31.8 (15.0-47.6)
1-4	184 (0.2)	2,944 (7.4)
5-9	1,305 (1.6)	2,984 (7.5)
10-19	5,253 (6.6)	7,528 (18.9)
20-29	7,647 (9.6)	5,927 (14.9)
>=30	65,592 (82.0)	20,470 (51.4)
**Chemotherapy**		
No chemotherapy	40,501 (50.6)	24,957 (62.6)
Chemotherapy >180 days pre surgery	1,539 (1.9)	215 (0.5)
Neoadjuvant chemotherapy (0-180 days pre surgery)	16,670 (20.8)	3,118 (7.8)
Adjuvant chemotherapy	17,062 (21.3)	10,169 (25.5)
Unknown chemotherapy or unknown chemotherapy date	4,209 (5.3)	1,394 (3.5)
**Radiation**		
No radiation	65,530 (81.9)	36,115 (90.6)
Radiation > 180 days before surgery	351 (0.4)	69 (0.2)
Neoadjuvant radiation (0-180 days before surgery)	9,840 (12.3)	2,040 (5.1)
Adjuvant radiation	3,579 (4.5)	1,474 (3.7)
Unknown radiation or unknown radiation date	681 (0.9)	155 (0.4)
**Surgical Margins**		
No residual tumor	73,807 (92.3)	37,182 (93.3)
Residual Tumor NOS	1,866 (2.3)	1,043 (2.6)

© 2020 Boffa DJ et al. *JAMA Network Open.*

Microscopic residual tumor	3,242 (4.0)	1,253 (3.1)
Macroscopic residual tumor	236 (0.3)	116 (0.3)
Margins not evaluable	259 (0.3)	95 (0.2)
Missing	571 (0.7)	164 (0.4)
**Unplanned 30-day readmission[10]**		
Yes	4,061 (5.1)	2,028 (5.1)
No	75,920 (94.9)	37,825 (94.9)
**% Compliance w/ CoC Quality Measures (95% CI)[11]**		
Colon cancer, *scenario-specific* use adjuvant Chemotherapy	89.0 (88.1-89.8)	89.9 (89.1-90.6)
Colon Cancer, ≥12 Regional Lymph nodes	95.1 (94.8-95.5)	91.5 (91.1-91.8)
Lung cancer, *scenario-specific* use adjuvant chemotherapy	92.5 (91.7-93.2)	93.2 (92.2-94.2)
Lung Cancer, >10 lymph nodes	50.7 (50.0-51.4)	45.6 (44.7-46.6)

IQR – Interquartile Range, CoC – Commission on Cancer, CI – Confidence Interval

1. Chi square p-value <.001 for all variables except unplanned readmission. This table contains patients in whom pathologic stage was missing (unlike Table 1 in body of manuscript, in which patients without pathologic stage were excluded).
2. Based on census zip code median income, 2012-2016 American Community Survey data.
3. Based on 2013 patient county and state and United States Department of Agriculture Economic Research Service Urban rural continuum codes at. https://www.ers.usda.gov/data-products/rural-urban-continuum- codes
4. Crow fly distance between the patient and hospital zip codes.
5. Only includes patients who were known to have gone directly to surgery (i.e. did not have neoadjuvant chemotherapy or radiation. N=56,917 for top-ranked hospital, 34,751 for affiliates. 6. AJCC 7th edition Clinical Stage IV and AJCC 7th edition clinical M+ excluded from the analyses.
7. Census division categories: New England: CT, MA, ME, NH, RI, VT; Middle Atlantic: NJ, NY, PA; South Atlantic: DC, DE, FL, GA, MD, NC, SC, VA, WV; East North Central: IL, IN, MI, OH, WI; East South Central: AL, KY, MS, TN; West North Central: IA, KS, MN, MO, ND, NE, SD; West South Central: AR, LA, OK, TX; Mountain: AZ, CO, ID, MT, NM, NV, UT, WY; Pacific: AK, CA, HI, OR, WA
8. Commission on Cancer Hospital Category types are described at: https://www.facs.org/quality-programs/cancer/coc/apply/categories
9. Average annual volume calculated for all procedures included in the analysis, including patients with missing 90-day mortality. Procedure specific volumes are listed in Supplemental Table 3
10. p=0.93
11. Commission on Cancer Quality measures evaluated https://www.facs.org/quality-programs/cancer/ncdb/qualitymeasures outlined below:
    - Adjuvant chemotherapy is recommended or administered within 4 months (120 days) of diagnosis for patients under the age of 80 with AJCC Stage III (lymph node positive) colon cancer.
    - At least 12 regional lymph nodes are removed and pathologically examined for resected colon cancer.
    - Systemic chemotherapy is administered within 4 months to day preoperatively or day of surgery to 6 months postoperatively, or it is recommended for surgically resected cases with pathologic, lymph node-positive (pN1) and (pN2) NSCLC.
    - At least 10 regional lymph nodes are removed and pathologically examined for AJCC stage IA, IB, IIA, and IIB resected NSCLC

© 2020 Boffa DJ et al. *JAMA Network Open.*

eTable 2. Annual Procedure Volumes

	Median[2]	Interquartile range
**All procedures**		
Top-ranked	68.2	37.8-111.4
Affiliate	31.8	15.0-47.6
**Esophagectomy**		
Top-ranked	25.0	14.6-39.8
Affiliate	4.0	2.3-5.8
**Gastrectomy**		
Top-ranked	30.8	23.0-45.0
Affiliate	6.3	3.6-10.8
**Colectomy**		
Top-ranked	86.8	58.2-127.8
Affiliate	39.8	27.0-57.4 26.8-57.4
**Proctectomy**		
Top-ranked	18.8	10.2-37.3
Affiliate	6.0	3.0-13.0
**Whipple**		
Top-ranked	42.6	29.4-83.8
Affiliate	9.2	4.8-19.25
**Lobectomy/Pneumonectomy**		
Top-ranked	103.4	72.4-162.0
Affiliate	26.4	15.0-43.8
**Cystectomy**		
Top-ranked	45.6	28.0-71.6
Affiliate	8.2	3.7-19.4

1. Volume based on all complex cancer procedures (as outlined in Methods) 2012-2016, including those missing 30 or 90 day mortality.

2. Two sided Wilcoxon p-values, all significant at <.001

© 2020 Boffa DJ et al. *JAMA Network Open.*

eTable 3. Time to Treat Across Different Procedures

Procedure and facility type	Median days to resection, N, any distance	P-value[2]
**Proctectomy**		
Top-ranked	42.0 (799) (IQR 23-67)	<.001
Affiliate	32.0 (401) (IQR 14-55)	
**Total Gastrectomy**		
Top-ranked	38.0 (795) (IQR 20-64)	0.02
Affiliate	33.0 (223) (IQR 12-52)	
**Partial Gastrectomy**		
Top-ranked	40.0 (2,568) (IQR 20-63)	<.001
Affiliate	21.0 (968) (IQR 0-42)	
**Total Colectomy**		
Top-ranked	22.0 (1,300) (IQR 0-46)	<.001
Affiliate	6.5 (642) (IQR 0-29)	
**Partial Colectomy**		
Top-ranked	23.0 (17,165) (IQR 6-40)	<.001
Affiliate	11.0 (20,363) (IQR 0-28)	
**Esophagectomy**		
Top-ranked	58.0 (1,028) (IQR 39-86)	0.01
Affiliate	49.0 (119) (IQR 28-70)	
**Lobectomy**		
Top-ranked	33.0 (18,806) (IQR 11-56)	<.001
Affiliate	31.0 (8,607) (IQR 3-52)	
**Pneumonectomy**		
Top-ranked	39.0 (1,063) (IQR 22-57)	0.19
Affiliate	35.0 (297) (IQR (19-55)	
**Whipple**		
Top-ranked	20.0 (6,756) (IQR 3-35)	<.001
Affiliate	15.0 (1,159) (IQR 0-29)	
**Cystectomy**		
Top-ranked	57.0 (4,821) (IQR 37-85)	<.001
Affiliate	49.0 (908) (IQR 29-76)	
**All Procedures**		
Top-ranked	30.0 (55,101) (IQR 10-53)	<.001
Affiliate	17.0 (33,687) (IQR 0-37)	

1. Excludes patients with missing 90-day mortality and missing pathologic stage. Excludes patients with neo-adjuvant chemotherapy or radiation or unknown neo-adjuvant radiation and chemotherapy.
2. Two-sided Wilcoxon test.

© 2020 Boffa DJ et al. *JAMA Network Open.*

eTable 4. Unplanned Readmission Within 30 Days

	Unplanned 30-day readmission percent	Total N	P-value[2]
**Proctectomy**			
Top-ranked	7.3	2,141	0.86
Affiliate	7.5	1,348	
**Total Gastrectomy**			
Top-ranked	5.9	1,377	0.87
Affiliate	6.1	427	
**Partial Gastrectomy**			
Top-ranked	4.6	3,514	0.62
Affiliate	4.9	1,385	
**Total Colectomy**			0.16
Top-ranked	6.8	1,026	
Affiliate	8.7	668	
**Partial Colectomy**			
Top-ranked	4.2	15,011	<.001
Affiliate	5.0	20,898	
**Esophagectomy**			0.82
Top-ranked	6.4	2,556	
Affiliate	6.6	466	
**Lobectomy**			0.03
Top-ranked	3.0	15,494	
Affiliate	3.6	8,731	
**Pneumonectomy**			0.22
Top-ranked	7.2	902	
Affiliate	5.3	342	
**Whipple**			0.19
Top-ranked	7.4	5,863	
Affiliate	8.6	1,190	
**Cystectomy**			0.38
Top-ranked	9.4	5,273	
Affiliate	8.7	1,487	
**All procedures**			0.78
Top-ranked	5.1	53,157	
Affiliate	5.1	36,942	

1. 29,735 patients excluded who traveled > 50 miles to resection facility.
2. Chi square p-value, Unplanned readmission vs no unplanned readmission.

© 2020 Boffa DJ et al. *JAMA Network Open.*

eAppendix. Supplemental Methods

General Study Overview

The study was submitted as a proposal to the Commission on Cancer, Quality Integration Committee. After review by members of the NCDB research team and senior members of the CoC, the study was approved as a project that had the potential to address a CoC mission to improve care for patients by studying a potential gap in safety. As is common for NCDB special studies (i.e. those that require additional detail than is provided in the Participant User File), a collaboration was formed between the principal investigator (Boffa), the NCDB research team, the CoC and the American College of Surgeons.

Additional Patient Selection Detail

The selected cancer procedures were chosen because they have been associated with a higher mortality rate, and likely involve multiple different teams of specialists. Furthermore, these procedures have been studied and reported multiple times under the category of “complex cancer surgeries”. While this cohort of procedures does not represent all the complex cancer procedures, we believe this comprises more than 80% of complex cancer surgeries routinely performed in the U.S.

Only cases in which the reporting hospital was also the hospital that performed the procedure were included.

Focus on US News and World Report Rankings

The USNWR rankings were selected for the cohort of hospitals that we anticipated to have better outcomes, that were affiliated with hospitals that have more opportunities to improve in terms of safety and potentially effectiveness. Our prior studies have shown that 1) the public believes that Top Ranking by USNWR is important to their choice, 2) affiliation with a top-ranked hospital increases their preference for care at the affiliate, 3) important differences in safety exist within these networks, and 4) these networks have a large market share of complex cancer surgery (1/3 of cancer surgeries takes place within these networks around the USNWR top 50 hospitals.

We recognize that additional metrics of superior quality could have been chosen (for example, designation as a National Cancer Institute NCI as a comprehensive cancer center). This would be another reasonable approach to take. It is worth noting that there is considerable cross over between the NCI comprehensive cancer centers and the top-ranked hospital cohort.

Additional Hospital Selection Detail

© 2020 Boffa DJ et al. *JAMA Network Open.*

Hospitals that were both in the top-50 *and* also affiliated with another top-ranked cancer hospital were all considered in the top-ranked cohort.

For affiliations that developed during the study period, only surgeries that occurred while the affiliation was in place were included in the analyses. The study was not designed to examine specific types of affiliations. However, two hospital mergers were identified during the study period between a top-ranked hospital and another hospital that was not ranked. The merged hospitals are considered together in the top-ranked cohort.

Overall 13 of the top-ranked hospitals had no CoC accredited affiliates. These 13 hospitals were included in the top-ranked cohort. Three of the top-ranked hospitals were not CoC accredited, however, 3 of their affiliate hospitals were. These 3 affiliates (of top-ranked hospitals who were not CoC accredited) were included in the affiliate cohort, though not the corresponding top-ranked hospital. A sensitivity analysis of 90-day mortality demonstrated no difference when the 13 top-ranked hospitals with no CoC accredited affiliates and the affiliates of non-CoC accredited top-ranked hospitals were excluded (OR 1.75 95%CI 1.54-1.99, p<.001).

De-identified Study-Set Creation

The list of top ranked hospitals and their brand-sharing affiliates was provided to the NCDB research team. The NCDB research team then queried the NCDB hospital identifiers for the eligible hospitals (i.e. were CoC accredited). The NCDB then compiled a de-identified database with an indicator for whether the hospital at which the patient underwent surgery was a top-ranked hospital, or the affiliate of a top-ranked hospital at the time surgery took place. This indicator was then used to consider these procedures in aggregate.

Variables

Age is shown as both a continuous and categorical variable. In order to account for the possibility that particularly advanced age strata (i.e. geriatric patients) could have disproportional risk, age was included as categorical variable. The NCDB only captures readmissions to the reporting hospital which performed the surgery. Greater travel distances may reduce the likelihood of patients being readmitted to the hospital that performed the surgery, therefore readmissions were examined exclusively in patients who traveled <50miles (90,099/119,834 patients)[2]

Clinical stage was missing in a substantial proportion of cases, more so in the patients treated at affiliate hospitals. This was primarily due to tumor extension being missing in a proportion of colon cancer clinical staging reports. As a sensitivity analysis, clinical stage was evaluated in all patients excluding colon, and rate of missing data was similar between top-ranked hospitals (Supplemental Table 1).

© 2020 Boffa DJ et al. *JAMA Network Open.*

Chemotherapy was considered as induction if given within 180 days of surgery. For 90-day mortality adjusted analyses, chemotherapy and radiation were considered in the following groupings: yes, none, unknown. Patients that received either chemotherapy more than 180 days prior to surgery, or those that received only adjuvant chemotherapy were considered as none for neoadjuvant.

"Time to treat" was only evaluated in patients that underwent surgery as their first treatment, as the induction strategy can be quite different in terms of number of cycles and duration. This variable is calculated internally by the NCDB. This variable is based off dates which are captured by the NCDB but are not publicly shared.

Extended surgical procedures, which involved removing portions of surrounding organs or tissues, were included in the analyses and adjusted for with an "extended" modifier. The inclusion of extended cases was important, as this could reflect surgical judgement with outcome implications. More specifically, if a patient had an invasive tumor that was growing close to surrounding tissues, the surgeon's decision to take additional tissue could influence prognosis (i.e. sparing continuous tissue could result in a close or positive margin).

Missing data

For the majority of variables that were included in adjusted models, rate of missing data was less than 5% (range 0 to 13.2%). Missing variables, other than pathologic stage, were imputed using multiple imputation technique. For 90-day mortality and long-term survival, only cases with complete pathologic stage data were considered. A complete case analysis was done for pathologic staging because: 1) the rate of missing data was low, 2) the complexity of imputing across different cancers and procedures while accounting for down-staging with induction therapy types may affect the reliability of imputation.

Statistics

The data were analyzed in aggregate, as opposed to comparing each top-ranked hospital, to each of its affiliates. This was done for multiple reasons. 1) We wished to show an average effect, which was representative of what was taking place across the many types of affiliations, hospital environments and populations in the United States. We recognize there are important nuances that could influence the relative safety and effectiveness of care that we cannot control for. As such, we feel that the aggregate approach best matched with the data that is available. 2) The surgical volumes were not sufficient to compare each hospital to each affiliate. Notably, all models accounted for correlation of outcomes within hospitals. 3) We performed a variance decomposition analysis to assess the proportion of variance in survival

© 2020 Boffa DJ et al. *JAMA Network Open.*

that was explained by patient, hospital and network levels; this found that 95.3% of the variance was at the patient level, 4.6% was at the hospital level, but only 0.1% was at the network level. This obviated accounting for correlation of outcomes within networks in our models, and also supported the overall design.

Kaplan Meier is shown as 3 -year survival, because the median duration of follow-up was felt to be more supportive of a shorter endpoint. 5-year survival statistics are shown as well in supplemental Table 2. Unadjusted survival is shown as this provides several important perspectives for clinicians. First this allows clinicians to view the outcomes of these cohorts in the context of other reported outcomes, including the AJCC staging manual (which describes unadjusted stage-specific survival). Second, this allows clinicians to view the shape of the curves for patterns in survivorship loss. Wide separation could represent surgical complications or health related differences, while cancer specific differences would be expected to impact curves a bit later in the postoperative course.

All analyses were at the patient level, with an indicator for affiliate vs top-ranked used to estimate the main study effect. For 90-day mortality outcomes we used mixed effects logistic regression models, estimating separate models for each procedure and a pooled model which included procedure indicators. To identify the functional form for the survival models, they were first estimated as Cox frailty models, and assessed for violations of the proportional hazards. Schoenfeld residual tests rejected the proportional hazards assumption,[3] therefore, we estimated a set of parametric time to event models, using Akaike Information Criteria (AIC) to identify the gamma distribution as providing the best fit[4]. Mixed effects gamma models for each cancer type included the same risk adjusters as for 90-day mortality, with additional indicators in the lung, colon, and gastric models for procedure type. The pooled model also included indicators for cancer type. For the survival models we report time ratios (TRs) which represent relative decrease in survival time for patients at affiliate hospitals.

Race is a variable that can track with the hospitals in which care is accessed, and can therefore confound the relationship between affiliation and outcomes. As such, race was not included in the primary adjusted models. However, recognizing that race may have some impact on both hospital selection and patient outcome, race/ethnicity were included in our propensity score models outlined below.

To account for potential selection bias, we estimated propensity models where the outcome was treatment at an affiliate hospital. We estimated separate models for the 90-day mortality and survival cohorts, adjusting each for all covariates in the main pooled models (including cancer type or procedure type, respectively), as well as race, area income level, and area educational level; these models were used to predict propensity scores for each patient in each

cohort. Because observation level weighting is problematic for mixed effects models, we incorporated these propensity scores as covariates in all models.

Age was examined for potential interactions. For age, the observed effect was particularly strong at advanced age, but no interactions were noted that undermined the main findings. Prior cancer history was included in the adjustment models (as opposed to simply excluding all patients with prior cancer history), per observations from our prior work[5].

REFERENCES

1. Resio BJ, Hoag JR, Chiu AS, et al. Variations in Surgical Safety According to Affiliation Status With a Top-Ranked Cancer Hospital. *JAMA Oncol.* 2019.
2. Turrentine FE, Buckley PJ, Sohn MW, Williams MD. Travel Time Influences Readmission Risk: Geospatial Mapping of Surgical Readmissions. *Am Surg.* 2017;83(6):573-582.
3. Schoenfeld D. Partial Residuals for the Proportional Hazards Regression Model. *Biometrika.* 1982;69(1):239-241.
4. Cox C, Chu H, Schneider MF, Muñoz A. Parametric survival analysis and taxonomy of hazard functions for the generalized gamma distribution. *Statistics in Medicine.* 2007;26(23):4352-4374.
5. Monsalve AF, Hoag JR, Resio BJ, et al. Variable impact of prior cancer history on the survival of lung cancer patients. *Lung Cancer.* 2019;127:130-137.

© 2020 Boffa DJ et al. *JAMA Network Open.*
